# Basic Predictive Risk Factors for Cytokine Storms in COVID-19 Patients

**DOI:** 10.3389/fimmu.2021.745515

**Published:** 2021-11-10

**Authors:** Sergey G. Shcherbak, Anna Yu Anisenkova, Sergei V. Mosenko, Oleg S. Glotov, Alexander N. Chernov, Svetlana V. Apalko, Stanislav P. Urazov, Evgeny Y. Garbuzov, Dmitry N. Khobotnikov, Olga A. Klitsenko, Evdokia M. Minina, Zakhar P. Asaulenko

**Affiliations:** ^1^ City Hospital №40, Saint Petersburg, Russia; ^2^ Department of Postgraduate Medical Education, Saint Petersburg State University, Saint Petersburg, Russia; ^3^ Federal State-Financed Institution Pediatric Research and Clinical Center for Infectious Diseases under the Federal Medical Biological Agency, Saint Petersburg, Russia; ^4^ Department of Pathphysiology, Institute of Experimental Medicine, Saint Petersburg, Russia; ^5^ North-Western State Medical University named after Ivan Ivanovich (I.I.) Mechnikov, Saint Petersburg, Russia

**Keywords:** COVID-19, cytokine storm, clinical and biochemical prognostic factors, patients, prognostic scale

## Abstract

**Objective:**

A critical role in coronavirus disease 2019 (COVID-19) pathogenesis is played by immune dysregulation that leads to a generalized uncontrolled multisystem inflammatory response, caused by overproduction of proinflammatory cytokines, known as “a cytokine storm” (CS), strongly associated with a severe course of disease. The aim of this study is to identify prognostic biomarkers for CS development in COVID-19 patients and integrate them into a prognostic score for CS-associated risk applicable to routine clinical practice.

**Materials and Methods:**

The authors performed a review of 458 medical records from COVID-19 patients (241 men and 217 women aged 60.0 ± 10.0) who received treatment in the St. Petersburg State Budgetary Institution of Healthcare City Hospital 40 (City Hospital 40, St. Petersburg), from Apr. 18, 2020 to Nov. 21, 2020. The patients were split in two groups: one group included 100 patients with moderate disease symptoms; the other group included 358 patients with progressive moderately severe, severe, and extremely severe disease. The National Early Warning Score (NEWS) score was used alongside with clinical assessment, chest computed tomographic (CT) scans, electrocardiography (ECG), and lab tests, like ferritin, C-reactive protein (CRP), interleukin (IL)-6, lactate dehydrogenase (LDH), and D-dimer.

**Results:**

The basic risk factors for cytokine storms in COVID-19 patients are male gender, age over 40 years, positive test result for replicative severe acute respiratory syndrome coronavirus 2 (SARS-CoV-2) RNA, absolute lymphocyte count, dynamics in the NEWS score, as well as LDH, D-dimer, ferritin, and IL-6 levels. These clinical and instrumental findings can be also used as laboratory biomarkers for diagnosis and dynamic monitoring of cytokine storms. The suggested prognostic scale (including the NEWS score dynamics; serum IL-6 greater than 23 pg/ml; serum CRP 50 mg/L or greater; absolute lymphocyte count less than 0.72 × 10^9^/L; positive test result for replicative coronavirus (SARS-CoV-2) RNA; age 40 years and over) is a useful tool to identify patients at a high risk for cytokine storm, requiring an early onset of anti-inflammatory therapy.

## Introduction

Coronavirus disease 2019 (COVID-19) infection caused by SARS-CoV-2 coronavirus remains a global challenge for the world’s healthcare system. Most people infected with SARS-CoV-2 will experience mild illness. Some patients, however, develop a dysregulated immune response, inducing severe lung injury. This is manifest as acute respiratory distress syndrome (ARDS) which precedes acute respiratory failure, extrapulmonary organ dysfunction, and high risk of death. COVID-19 disease is usually associated with elevated inflammatory biomarkers, cytokines, and chemokines, especially in severe cases. Moreover, lymphocytopenia and neutrophilia often emerged, with particularly declined counts of CD8+ T cells, CD4+ Т cells, and natural killer (NK) cells (1). The inpatient death rate varies between 15% and 20% or even higher among those requiring ICU admission (2). A critical role in COVID-19 pathogenesis is played by immune dysregulation that leads to a generalized uncontrolled multisystem inflammatory response, caused by overproduction of proinflammatory cytokines, known as “a cytokine storm” (CS). The CS manifestations are fever, cytopenia, hyperferritinemia, abnormal levels of liver transaminases, coagulopathy, and lung damage (including ARDS) (3). In all these conditions, cytokines interleukin (IL)-1β, IL-18, IFN-γ, and IL-6 are the key mediators responsible for the hyperinflammatory state. COVID-19-associated CS is a unique form of hyperinflammatory response, requiring further characterization criteria (4). The aim of this study is to identify prognostic biomarkers for CS development in COVID-19 patients and integrate them into a prognostic score for CS-associated risk applicable to routine clinical practice.

## Materials and Methods

### Study Design and Inclusion Criteria

The study design is an observational clinical trial. We utilized 458 medical records from COVID-19 patients who received treatment in the St. Petersburg State Budgetary Institution of Healthcare City Hospital 40 (City Hospital 40, St. Petersburg) from Apr. 18, 2020 to Nov. 21, 2020; the patients tested positive for SARS-CoV-2 RNA by polymerase chain reaction (PCR) amplification of nucleic acids from clinical material and presented clinical manifestations and symptoms (fever, general fatigue, malaise, cough, and dyspnea), features of viral pneumonia seen on unenhanced lung CT scan (noted as multiple lobular abnormalities often located in the peripheral areas of the lower lobes and manifested with predominantly perivascular bilateral disease distribution; multiple peripheral areas of ground-glass opacities with rounded morphology and variable extent; interlobular septal thickening/flattening that causes a crazy-paving pattern, areas of consolidation, air bronchogram sign, etc.) (5, 6).

### Characteristics of Groups of Patients

**Table 1** shows demographic characteristics, history, and comorbidities of the patient study cohort.

**Table 1:** Demographic characteristics, history, and comorbidities of the patient study cohort.

Parameter	*n* (%)
Age (years)	
≤39	38 (8.30%)
40–49	58 (12.66%)
50–59	123 (26.86%)
60–69	139 (30.35%)
≥70	100 (21.83%)
History of exposure to infected individuals	100 (22.22%)
History of travel outside the place of residence during the past 14 days	45 (9.83%)
Flu-resembling in relatives, including fever, cough, fatigue	44 (9.61%)
History of other diseases	
Hypertension	260 (56.77%)
Coronary artery disease	222 (48.47%)
Cerebrovascular disease	139 (30.35%)
Poststroke condition	97 (21.18%)
Condition following acute myocardial infarction	34 (7.42%)
Condition following a surgical intervention	89 (19.43%)
Rheumatoid arthritis and other autoimmune diseases	65 (14.19%)
Diabetes mellitus	63 (13.76%)
Chronic kidney disease, stages 3 to 5	32 (6.99%)
Malignant lesions	22 (4.80%)
Chronic obstructive pulmonary disease	20 (4.37%)
Chronic bronchitis	20 (4.37%)
Chronic asthma	13 (2.84%)

In accordance with the International and Russian Recommendations for the Prevention, Diagnosis and Treatment of New Coronavirus Infection (COVID-19), all patients were divided in two groups of comparable age (7, 8). The first group included 100 (21.8%) patients with clinical and radiological characteristics of stable, moderately severe course of disease. The criteria for an easy course were considered to be body temperature below 38°C, cough, weakness, sore throat, and the absence of criteria for moderate and severe course. The criteria for a moderate course are fever, temperature above 38°C, respiratory rate over 22/min, shortness of breath during exercise, pneumonia (exposed to CT of the lungs), and SpO_2_ <95%. The second group included 358 (78.2%) patients with progressive, moderately severe, severe, and extremely severe course of disease. Clinical and radiological criteria for severe course were respiratory rate more than 30/min, SpO_2_ 93%, PaO_2_/FiO_2_ 300 mmHg, progression of changes in the lungs typical for COVID-19 pneumonia according to CT data, including an increase in the prevalence of revealed changes by more than 25%, as well as the appearance of signs of other pathological conditions, changes in the level of consciousness, unstable hemodynamics (systolic blood pressure less than 90 mmHg or diastolic blood pressure less than 60 mmHg, urine output less than 20 ml/h), and qSOFA >2 points. The criteria for an extremely severe course were signs of ARF with the need for respiratory support (invasive ventilation), septic shock, and multiple organ failure. Thus, patients with mild and moderate and severe and extremely severe course are shown in **Table 2**.

**Table 2:** Disease severity in different groups of patients.

Parameter	Group 1		Group 2		Total	*p*-value
	*n*	%	*n*	%		
Males	58	58.0	159	44.4	217	0.016
Females	42	42.0	199	55.6	241	
Moderate severity of disease						
Moderate	100	100.00	153	42.74	253	0.000
Severe and extremely severe	0	0.00	205	57.26	205	
Lung involvement evaluation at admission based on a 4-point CT score ranking						
CT-1	57	57.0	82	22.9	139	
CT-2	43	43.0	223	62.3	263	0.000
CT-3	0	0.0	44	12.3	47	
CT-4	0	0.0	9	2.5	9	
Outcomes						
Survivals	100	100.0	255	71.2	355	0.000
Deaths	0	0.0	103	28.8	103	

Assessment of the severity of changes in the lungs (volume, area, length) in patients with COVID-19 pneumonia was carried out visually on the basis of computer programs for assessing the volume of compacted lung tissue and mapping the density of the pulmonary parenchyma in both lungs: (1) Absence of characteristic manifestations (CT-0). (2) Minimum volume/prevalence <25% of lung volume (CT-1). (3) Average volume/prevalence 25%–50% of lung volume (CT-2). (4) Significant volume/prevalence of 50%–75% of lung volume (CT-3). (5) Critical volume prevalence >75% of lung volume (CT-4) (9).

Symptoms at admission were fever in 365 (80%) patients, cough in 329 (72%) patients, dyspnea in 265 (57.86%) patients, myalgia in 43 (9.39%) patients, general fatigue in 344 (75.11%) patients, headache in 36 (7.86%) patients, a sore throat in 29 (6.33%) patients, a runny nose or rhinorrhea in 46 (10.04%) patients, chest pain in 51 (11.14%) patients, diarrhea in 34 (7.42%) patients, nausea and vomiting in 13 (2.84%) patients, and impaired taste and smell in 40 (8.73%) patients. Four hundred fifty (98.25%) reported one or more disease-specific symptoms. CT imaging manifestation of pneumonia was detected in 458 (100%) patients.

### Clinical and Biochemical Surveillances

Medical examination of all patients included history taking, with a focus on the course of disease, physical examination, and assessment of hemodynamics and respiratory system (respiratory rate, heart rate, blood pressure, SpO_2_, respiratory distress); calculation of the National Early Warning Score (NEWS), a recommended scoring system for use in COVID-19 patients (10, 11); computed tomography (CT) of the chest with the severity score ranking on a 4-point scale (CT-1, CT-2, CT-3, CT-4); laboratory tests (complete haemogram, basic blood chemistry panel, ferritin test, С-reactive protein, IL-6, lactate dehydrogenase test, D-dimer); ECG; and other instrumental examinations, if required.

### Therapy for Patients With COVID-19 Infection

In group 1, treatment of COVID-19 and its complications included antibacterial and antiviral drugs, prevention of hypercoagulability and disseminated intravascular coagulation, symptom-related treatment, and oxygen therapy. To prevent or treat the cytokine storm depending on the disease severity, in group 2, standard treatment was supplemented with convalescent plasma therapy, anticytokine drugs: interleukine-6 (IL-6) inhibitors (tocilizumab, olokizumab, levilimab), IL-1 inhibitors (canakinumab, RH104), JAK inhibitors (tofacitinib, ruxolitinib, baricitinib), Bcr–Abl tyrosine kinase inhibitor (radotinib), and glucocorticoids for some cases. Respiratory therapy, modified antibacterial therapy, extracorporeal membrane oxygenation, sepsis, and septic shock treatment (extracorporeal detoxication and blood purification, etc.) were performed in a stepwise fashion according to indications (5).

### Statistical Analysis

Data were analyzed using STATISTICA for Windows (version 10, license No. BXXR310F964808FA-V) software. Since all data (except age) did not follow a normal distribution, we applied the Mann-Whitney, Kolmogorov-Smirnov, median Chi-square, and ANOVA tests to evaluate between-group differences in quantitative parameters (age, NEWS score, D-dimer, **C**-reactive protein (CRP), IL-6, etc.) and normality of sampling distribution. We used Pearson’s Chi-squared and Fischer’s exact nonparametric tests to assess the frequencies of qualitative data (gender, disease manifestations and severity, complaints). Classification Trees were utilized to identify cutoff thresholds for age, NEWS score, and lab test values. The odds ratio (OR) for developing the CS—the ratio of probabilities of a certain event for different groups—was calculated for a 2 × 2 contingency table using a standard formula and the associated confidence interval. We applied the Haldane’s correction, if the 2 × 2 table contained a zero cell.

## Results

### Comparison of Clinical and Lab Variables in Different Groups of Patients

At admission, group 1 patients showed a reliably higher frequency of chest CT score 1, whereas group 2 patients were admitted with more extensive lung involvement (CT score ranging from 2 to 4). Although moderately severe lung involvement (CT score 2) prevailed in group 2, at admission, these patients reported manifestations of progressive respiratory failure and fever (**Table 3**). There is a reliable difference in the NEWS score: group 1 had the average admission NEWS score of 2 and the average hospital stay of 11 days; group 2 had the average NEWS score of 4 at admission which exacerbated to 5 at the start of treatment, including anticytokine drugs, anti-COVID-19 convalescent plasma, and hemoadsorbtion; the average hospital stay in group 2 was 12 days. The death rate due to comorbidities was highest in group 2 patients with severe and extremely severe course of disease (28.8% in group 2 and 22.5% in the total cohort). At baseline, such patients had an unfavorable forecast due to age, comorbidity, clinical severity of acute respiratory failure, a high NEWS score, dynamic evaluation of extensive lung involvement, and exacerbation of lung injury based on chest CT (**Table 3**). The virus-induced infection elicits inflammatory manifestations (absolute lymphocyte count and levels of LDG, CRP, ferritin, D-dimer, and IL-6) that correlate with a CS scenario (lymphocytopenia, hypercytokinemia, hyperinflammation) (12).

**Table 3:** Between-group differences in NEWS score, time from symptoms onset to hospital admission, and length of hospital stay.

Parameter	Characteristics	Group 1		Group 2		*p*-value
		*n*	Value	*n*	Value	
NEWS score at admission	M ± SD	100	2.4 ± 1.7	356	4.5 ± 2.7	<0.001
	min ÷ max		0 ÷ 8		0 ÷ 14	
NEWS score by the start of CS treatment	M ± SD	100	1.5 ± 1.6	357	5.68 ± 2.82	<0.001
	min ÷ max		0 ÷ 6		0 ÷ 14	
NEWS score at discharge	M ± SD	100	0.2 ± 1.02	349	3.29 ± 5.42	<0.001
	min ÷ max		0 ÷ 9		0 ÷ 16	
Days from symptoms onset to hospital admission	M ± SD	100	8.8 ± 5.9	356	6.63 ± 5.39	<0.001
	min ÷ max		0 ÷ 37		0 ÷ 57	
Day of disease on which CS treatment started (anticytokine drugs, convalescent plasma, hemoadsorbtion)	M ± SD	100	9.0 ± 6.0	357	10.35 ± 5.98	<0.017
	min ÷ max		1 ÷ 37		1 ÷ 59	
Length of hospital stay (days in hospital)	M ± SD	100	11.8 ± 4.9	355	13.6 ± 6.7	<0.012

The NEWS2 is based on a simple aggregate scoring system in which a score is allocated to six physiological measurements (respiration rate, oxygen saturation, systolic blood pressure, pulse rate, level of consciousness) when patients present to, or are being monitored in hospital.

Respiratory rate, per minute (NEWS score): ≤8 (+3), 9–11 (+1), 12–20 (0), 21–24 (+2), ≥25 (+3). Oxygen saturations, % (NEWS score): ≤91% (+3), 92%–93% (+2), 94%–95% (+1), ≥96% (0). Temperature, °C/°F (NEWS score): ≤35°C/95°F (+3), 35.1°–36°C/95.1°F–96.8°F (+1), 36.1°C–38°C/96.9°F–100.4°F (0), 38.1°C–39°C/100.5°F–102.2°F (+1), ≥39.1°C/102.3°F (+2).

Systolic blood pressure, mmHg (NEWS score): ≤90 (+3), 91–100 (+2), 101–110 (+1), 111–219 (0), ≥220 (+3).

Pulse rate, per minute (NEWS score): ≤40 (+3), 41–50 (+1), 51–90 (0), 91–110 (+1), 111–130) (+2). ≥131 (+3);

Consciousness: (NEWS score): alert (0), voice, pain, unresponsive (+3) (11).

In addition, prevalence of comorbidities in our cohort was greater than reported by other authors for adult patients with COVID-19 (31%) (13). The high rate of comorbidities in our patients is due to the specific profile of our department, which focuses on patients with a severe and extremely severe course of disease. Two hundred twenty-one (48%) patients were admitted to ICU from other departments or hospitals due to COVID-19 progression.

### Bioinformatic Analysis of Patient Data and Critical CS-Related Clinical, Instrumental, and Lab Characteristics

Comparative analysis of clinical, imaging, and lab characteristics in recruited cohorts of patients has revealed most critical CS-related variables (**Table 4**).

**Table 4:** Comparative analysis of basic variables for CS diagnosis at the onset of proactive anti-inflammatory treatment.

Parameter	Group 1		Group 2		*p*-value
	*n*	M ± SDmin÷max	*n*	M ± SDmin÷max	
Age (year)	100	57.53 ± 15.06	358	60.5 ± 13.37	0.05
		21 ÷ 86		24 ÷ 89	
Serum lymphocytes (10^9^/L)	98	1.49 ± 0.59	349	1.28 ± 1.39	<0.01
		0.46 ÷ 3.2		0.23 ÷ 24.62	
Serum LDH (U/L)	27	357.78 ± 155.3	149	410.17 ± 191.24	<0.1
		169 ÷ 914		134 ÷ 1492	
Serum CRP (mg/L)	91	54.61 ± 64.92	346	106.71 ± 79.58	<0.001
		0.5 ÷ 274.9		0.8 ÷ 361.9	
Serum ferritin (ng/ml)	20	328.57 ± 185.15	190	696.28 ± 792.88	<0.01
		57.1 ÷ 781.3		0 ÷ 7759.4	
Serum D-dimer (μg/ml)	29	1.26 ± 2.75	147	1.84 ± 2.79	<0.05
		0.27 ÷ 15.34		0.15 ÷ 18.69	
Serum IL-6 (pg/ml)	65	15.02 ± 23.64	318	161.26 ± 442.5	<0.001
		0 ÷ 127.2		1.5 ÷ 4894	
NEWS score dynamics from admission to the onset of CS treatment	100	−0.96 ± 1.19	356	1.24 ± 1.86	<0.001
		–4 ÷ 4		–3 ÷ 11	

The NEWS score dynamics across different patient groups exhibits qualitative differences: the NEWS score decreased from baseline in group 1 (by −1 [−2; 0] points) and increased in group 2 with progressive course of disease (by +1 [0; 2] points) (*р* < 0.001). There are reliable between-group differences in lab variables (absolute lymphocyte count, CRP, ferritin, D-dimer, IL-6), which correlate with the NEWS score dynamics at admission until the start of CS treatment.

Classification Trees were utilized to identify cutoffs for CS-associated risk factors (**Table 5**).

**Table 5:** Cutoffs for predictive risk factors of CS in groups 1 and 2 at the onset of proactive anti-inflammatory treatment.

Parameter	Group 1		Group 2		Total	*p*-value
	*n*	%	*n*	%	*n*	
Serum LDH (U/L)						
≤390	20	19.80	81	80.20	101	<0.1
>390	7	9.33	68	90.67	75	
Age						
<40 years	16	42.11	22	57.89	38	<0.01
≥40 years	84	20.00	336	80.00	420	
Test for replicative SARS-CoV-2 RNA						
Negative	39	43.82	50	56.18	89	<0.001
Positive	53	18.28	237	81.72	290	
Serum CRP (mg/L)						
<50	56	38.10	91	61.90	147	<0.001
≥50	35	12.07	255	87.93	290	
Serum lymphocytes (10^9^/L)						
≥0.72	94	25.47	275	74.53	369	<0.001
<0.72	4	5.13	74	94.87	78	
Serum D-dimer (μg/ml)						
≥2.1	28	19.44	116	80.56	144	<0.05
<2.1	1	3.13	31	96.88	32	
Serum ferritin (ng/ml)						
≥485	18	15.93	95	84.07	113	<0.01
<485	2	2.06	95	97.94	97	
NEWS score (points)						
<0	62	74.70	21	25.30	83	<0.001
≥0	38	10.19	335	89.81	373	
Serum IL-6 (pg/ml)						
≤23	54	52.94	48	47.06	102	<0.001
>23	11	3.91	270	96.09	281	

The table shows the threshold values for all parameters that are highlighted as borderline for the risk of developing a cytokine storm.

Group 2 patients showed a reliably higher frequency of prognostic variables exceeding CS-associated cutoff values (**Table 6**).

**Table 6:** CS incidence rate depending on the number of risk factors.

No. of CS-associated risk factors	Group 1		Group 2		Total
	*n*	%	*n*	%	
None	2	100.00	0	0.00	2
1 factor	12	100.00	0	0.00	12
2 factors	14	63.64	8	36.36	22
3 factors	21	37.50	35	62.50	56
4 factors	6	9.68	56	90.32	62
5 factors	2	1.64	120	98.36	122
6 factors	0	0.00	34	100.00	34
Total	57	18.39	253	81.61	310

This was followed by a comprehensive evaluation of CS-associated risk based on the ranking of variables obtained at admission. These variables were ranked by prognostic relevance in accordance with Classification Trees by using CART-style method for Split selection and included the following: NEWS score dynamics, serum IL-6 greater than 23 pg/ml, serum CRP 50 mg/l and greater, absolute lymphocyte count less than 0.72 × 10^9^/L, positive test for replicative SARS-CoV-2 RNA, and age 40 years and over. An assessment of the effectiveness of the obtained criteria was carried out, which confirmed the possibility of their application in clinical practice. These biomarkers can be used as predictive criteria to determine the CS risk. We should note that there are no meaningful gender-related differences in the comprehensive examination of CS-related risk.

**Figure 1:**
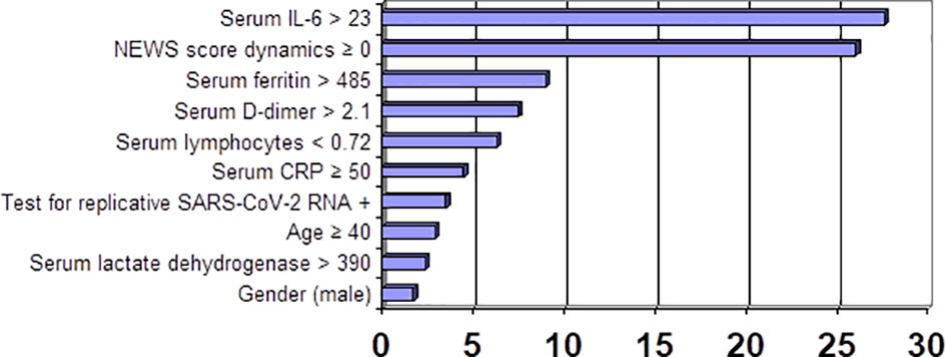
Incremental CS-associated risk (OR) and its correlation with unfavorable lab test values.

**Figure 1** shows incremental CS-associated risk (OR) and its correlation with lab test values.

**Figure 2:**
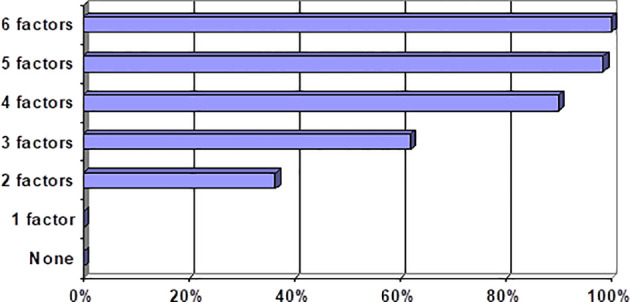
CS incidence rate in correlation with different sets of risk factors.

Increased CS incidence is associated with a larger number of risk factors (correlation coefficient Rg = +0.91, *р* < 0.001) (**Table 6**; **Figure 2**). Each of these factors, if combined with the largest number of other risk factors, exacerbated the CS-associated risk.

We outlined the following risk categories of patients to enhance clinical relevance of our prognostic model: category 1 (0 to 1 risk factors) = CS-associated risk is near zero; category 2 (two to three risk factors) = CS-associated risk escalates dramatically to 55%, augmenting 35.5-fold vs. category 1; category 3 (four and more risk factors) = CS-associated risk exacerbates to 96%, augmenting 718-fold vs. category 1. The obtained results are consistent with findings by other authors on the evaluation of risk factors for COVID-19-associated CS (14, 15). Our results provide a justification for a treatment strategy with an early onset of proactive anti-inflammatory therapy and anti-COVID-19 convalescent plasma in patients at a high risk for CS development.

## Discussion

Considering there are no reliable prognostic criteria for COVID-19-induced CS available today, we selected 458 patients with a different course of disease to evaluate prognostic power of available clinical, instrumental, and lab parameters; the most useful parameters for assessing outcome were collated into coherent domains, or clusters, and their prognostic power was evaluated. The clinical data collected from patient histories and at hospital admission included signs and symptoms, demographic, epidemiological, and clinical parameters, assessment of disease severity using the NEWS score, severity of COVID-19, comorbidities, dynamic evaluation of lung involvement (ground-glass opacities ± areas of consolidation) based on a standard imaging protocol for unenhanced chest CT (5), serum lab test values (16) recorded within 24 h prior or after the diagnosis of CS and over the next 7 days of hospital stay. Test results for replicative SARS-CoV-2 RNA, length of hospital stay and outcomes were assessed over the next 10 days. Potential risk factors for CS development were identified by comparing between patients with and without CS-associated clinical and CT manifestations. A deteriorating NEWS score is associated with a clinically severe disease and progressive hemodynamic impairment. Thus, in group 1 patients, admission NEWS score was no greater than 4, improving by 1–2 points after the onset of treatment; in contrast, the NEWS score was higher in group 2 patients at baseline, exacerbating further by 1.24 ± 1.86 points. Groups 1 and 2 presented reliable differences in IL-6, CRP, ferritin, and lymphocyte count values. In conclusion, our findings show that a progressive course of disease is associated with escalating values of biomarkers that induced the CS-associated clinical scenario in recruited patients.

## Conclusions

1. The basic risk factors for cytokine storms in COVID-19 patients include male gender, lactate dehydrogenase level, age over 40 years, positive test result for replicative SARS-CoV-2 RNA, absolute lymphocyte count, D-dimer and ferritin levels, dynamics in the NEWS score, and plasma IL-6 concentration.
2. Absolute lymphocyte count, LDH, CRP, ferritin, D-dimer, and IL-6 levels are the most critical lab parameters for diagnosis and dynamic monitoring of cytokine storms.
3. The suggested prognostic scale (including the NEWS score dynamics; serum IL-6 greater than 23 pg/ml; serum CRP = 50 mg/L or greater; absolute lymphocyte count less than 0.72 × 10^9^/L; positive test result for replicative coronavirus (SARS-CoV-2) RNA; age 40 years and over) is a useful tool to identify patients at a high risk for cytokine storm, requiring an early onset of anti-inflammatory therapy.

## Data Availability Statement

The original contributions presented in the study are included in the article/**Supplementary Material**. Further inquiries can be directed to the corresponding author.

## Ethics Statement

The studies involving human participants were reviewed and approved by City Hospital No. 40, Saint Petersburg. The patients/participants provided their written informed consent to participate in this study.

## Author Contributions

SS: concept development and research; guidance, choice of treatment strategy, and patient assessment; and analysis of the studied groups. AA, EG, and DK: treatment and examination of patients. SM and SU: literary search. AA and SM: formation of the base of the studied groups, statistical analysis, and writing an article. OK: statistical analysis. EM, ZA, and OG: performing X-ray, pathomorphological diagnostics and testing biological samples of patients for the presence of SARS-CoV-2 coronavirus. OG and AC: editorial of the article. SA: biobanking of materials. All authors contributed to the article and approved the submitted version.

## Funding

The research was financed from the budget of the St. Petersburg State Budgetary Institution of Healthcare City Hospital 40 and a grant from the funds of St. Petersburg State University ID PURE: 75253103.

## Conflict of Interest

The authors declare that the research was conducted in the absence of any commercial or financial relationships that could be construed as a potential conflict of interest.

## Publisher’s Note

All claims expressed in this article are solely those of the authors and do not necessarily represent those of their affiliated organizations, or those of the publisher, the editors and the reviewers. Any product that may be evaluated in this article, or claim that may be made by its manufacturer, is not guaranteed or endorsed by the publisher.
