# Supplementary material for: Basic Predictive Risk Factors for Cytokine Storms in COVID-19 Patients
Source: Front Immunol. 2021 Nov 10;12:745515. doi: 10.3389/fimmu.2021.745515 (PMC8631447; doi:10.3389/fimmu.2021.745515)
Supplement: Supplementary file 1 [file DataSheet_1.docx]

# Table 1. Demographic characteristics, history, and comorbidities of the patient study cohort.

| Parameter | Group 1 | Group 2 | All (%) |
| --- | --- | --- | --- |
| Quantity | 100 | 358 | 458 |
| Gender (men) | 42 (42%) | 199 (55.6%) | 241 |
| Gender (women) | 58 (58%) | 159 (44.4%) | 217 |
| Age, years | 57.5 | 60.5 | 59.8 |
| ≤39 | 16 (16%) | 22 (6.1%) | 38 (8.30%) |
| 40–49 | 9 (9%) | 49 (13.7%) | 58 (12.66%) |
| 50–59 | 25 (25%) | 98 (27.4%) | 123 (26.86%) |
| 60–69 | 30 (30%) | 109 (30.4%) | 139 (30.35%) |
| ≥70 | 20 (20%) | 80 (22/3%) | 100 (21.83%) |
| History of other diseases: | | | |
| Hypertension | 29 (29%) | 231 (64.5%) | 260 (56.77%) |
| Coronary artery disease | 100 (100%) | 122 (34.1%) | 222 (48.47%) |
| Cerebrovascular disease | 33 (33%) | 106 (29.6%) | 139 (30.35%) |
| Post stroke condition | 14 (14%) | 83 (23.2%) | 97 (21.18%) |
| Condition following acute myocardial infarction | 8 (8%) | 26 (7.2%) | 34 (7.42%) |
| Condition following a surgical intervention | 16 (16%) | 73 (20.4%) | 89 (19.43%) |
| Rheumatoid arthritis and other autoimmune diseases | 8 (8%) | 57 (15.9%) | 65 (14.19%) |
| Diabetes mellitus | 16 (16%) | 47 (13.1%) | 63 (13.76%) |
| Chronic kidney disease, stages 3 to 5 | 3 (3%) | 29 (8.1%) | 32 (6.99%) |
| Malignant lesions | 2 (2%) | 20 (5.6%) | 22 (4.80%) |
| Chronic obstructive pulmonary disease | 6 (6%) | 14 (3.9%) | 20 (4.37%) |
| Chronic bronchitis | 2 (2%) | 18 (5.0%) | 20 (4.37%) |
| Chronic asthma | 3 (3%) | 10 (2.8%) | 13 (2.84%) |
| Charlson Comorbidity Index, overall score | 3.7 | 4.6 | 4.4 |
